# Optimization of Antibiotic and Antimycotic Compositions for the Eradication of Microorganisms from Cell Cultures

**DOI:** 10.32607/actanaturae.27907

**Published:** 2026

**Authors:** Y. A. Prokopenko, E. A. Faizullina, O. V. Serova, S. V. Protsenko, Yu. A. Mokrushina, N. Gong, H. Zheng, Yu. B. Kuzmin, P. V. Tsarapaev, D. M. Malabuiok, Y. R. Vereveyko, I. V. Smirnov, N. E. Kushlinskii, H. Zhang, A. G. Gabibov, S. S. Terekhov

**Affiliations:** Shemyakin–Ovchinnikov Institute of Bioorganic Chemistry of the Russian Academy of Sciences, Moscow, 117997 Russia; State Key Laboratory of Medicinal Chemical Biology and College of Life Sciences, Nankai University, Tianjin, 300071 China; N.N. Blokhin National Medical Research Center of Oncology of the Ministry of Health of the Russian Federation, Moscow, 115522 Russia; Endocrinology Research Center, Ministry of Health of the Russian Federation, Moscow, 117036 Russia; Faculty of Chemistry, Lomonosov Moscow State University, Moscow, 119991 Russia

**Keywords:** cell cultures, mycoplasma, antibiotics, antimycotics, contamination, microorganisms

## Abstract

Cell cultures underlie the vast majority of *in vitro* research. Despite strict sterility requirements and the availability of high-tech equipment to prevent contamination, laboratory practice remains associated with a significant risk of contamination of mammalian cell cultures with bacterial and fungal agents. According to the American Type Culture Collection (ATCC), 15–35% of cell lines are contaminated with mycoplasma, posing a high risk of cross-contamination and leading to significant distortions of the experimental data obtained using such cultures. Furthermore, microbial contamination is a major risk factor when working with cell cultures isolated from patient surgical biospecimens. Despite the availability of ready-made solutions for eradicating microorganisms and preventing cell culture contamination, the problem of antibiotic resistance is particularly relevant for such contamination, and commercially available agents are often ineffective. In this study, combinations of antimicrobial agents were selected to address two of the most common practical challenges in cell culture: (i) complete elimination of mycoplasma infection and (ii) preventing contamination of primary cell cultures with bacteria and fungi that are components of the patient microbiome. A combination of tylosin, tetracycline, ciprofloxacin, erythromycin, and chloramphenicol was shown to completely eradicate mycoplasma from infected cell cultures. A combination of levofloxacin, polymyxin B, vancomycin, meropenem, and the antifungals micafungin and voriconazole completely eliminated the risk of microbial contamination during primary culture isolation, including from complex specimens such as surgical resections from colorectal cancer patients. The selected compositions successfully combine low cytotoxicity with a broad spectrum of antimicrobial activity, allowing for their effective use in laboratory practice. Nonetheless, the principle of minimizing the use of antimicrobial agents should be strictly adhered to in order to control the spread of antibiotic resistance.

## INTRODUCTION

Cell cultures underlie contemporary *in vitro* research [1, 2]. Studies in the fields of molecular and cellular biology [5], cytotoxicity assessment [6, 7], and numerous functional assays [8] would be impossible without standardized cell lines and various cell cultures [3, 4]. Maintaining the absence of microbial contamination is a fundamental principle of cell culture techniques [9]. Nevertheless, assessments by such a reputable organization as the American Type Culture Collection (ATCC) demonstrate that 15–35% of cell lines sourced from culture collections are contaminated with mycoplasma^1^. Even the lower bounds of the mycoplasma contamination rate estimated using the culture-independent genomic data indicate that over 10% of rodent and primate cell cultures are contaminated with mycoplasma [10]. According to the data from the German Collection of Microorganisms and Cell Cultures GmbH (DSMZ), approximately 28% of all lymphoma cell lines in culture collections are contaminated with mycoplasma [11]. In some collections, this figure can reach as high as 70% [12].

^1^Mycoplasma Contamination // American Type Culture Collection : [website]. 2012. URL: https://www.atcc.org/the-science/authentication/mycoplasma-contamination (accessed: 10.04.2026).

If a cell culture is contaminated with mycoplasma, the most practical solution is often to discard all associated biospecimens and perform extensive decontamination of the laboratory equipment. However, approaches based on targeted microbial eradication can be applied in certain cases when the contaminated culture holds exceptional value. The efficacy and toxicity profiles of currently available solutions and reagents for mycoplasma elimination are often suboptimal. Moreover, the growing challenge of antibiotic resistance is particularly relevant for contaminated cell cultures, thus hindering the complete eradication of intracellular mycoplasma infections [13].

Studies involving primary cell cultures sourced from microbiome-associated donor tissues are another important example of the critical need for complete microbial eradication. Such challenges are commonly associated with studies involving the isolation of primary cultures of lung cancer and colorectal cancer (CRC) in particular [14, 15, 16]. The conventional supplements for growth media, including the Gibco™ antibiotic–antimycotic mix (100X), lack sufficient efficacy, largely due to the increasing prevalence of resistant microbial strains.

Therefore, the development of more effective agents for microbial eradication from cell cultures poses an increasingly pressing challenge. In this study, we have addressed the two most common problems: (i) mycoplasma eradication and (ii) prevention of contamination of the primary cell cultures with bacteria and fungi that are components of the donor patient’s microbiome. The selection of the antibiotic and antimycotic composition for mycoplasma elimination was based on data on the efficacy of various antibiotics against bacteria of the genus *Mycoplasma* and the antimicrobial resistance of isolated bacterial strains [13]. The optimal composition for preventing primary cell culture contamination with bacteria and fungi was developed using a panel of the most effective antibiotics and antimycotics used in clinical practice. This composition was designed to include antibiotics belonging to different classes (fluoroquinolones, lipopeptides, glycopeptides, carbapenems, and tetracyclines) as well as varying in their spectrum of activity (polymyxins for the eradication of Gram-negative microorganisms, and glycopeptides for Gram-positive microorganisms), including highly potent antifungal agents. The selected compositions ensured complete eradication of mycoplasma infection and prevented bacterial and fungal contamination in samples isolated from surgical specimens.

## EXPERIMENTAL

**Cell cultures**

Primary cell cultures isolated from 38 colorectal cancer patients who had been receiving diagnostic evaluation and treatment at the “N.N. Blokhin National Medical Research Center of Oncology” of the Ministry of Health of the Russian Federation (NN Blokhin NMRCO) were included in the study. All the procedures conducted in the study complied with the guidelines of the institutional ethics committee and the 1964 Declaration of Helsinki, with its subsequent amendments or equivalent ethical standards. Each study subject provided a written informed consent. In all the patients, clinical diagnosis was verified by tumor morphology data according to the WHO Classification of Tumours of the Digestive System (WHO, 2019). Primary cell cultures were isolated using the procedure developed earlier [14].

The local collection of HEK-293T, A172, A431, U87, U251, HT-1080, and NCI-H1299 cell lines was originally sourced from ATCC. The effectiveness of mycoplasma eradication was evaluated for the samples contaminated with mycoplasma due to inadequate laboratory practices. The original master cell banks remained sterile and were used as controls.

**Isolating cells from the tumor**

Primary tumor cell cultures were isolated according to the protocol described previously [14]. A tumor fragment sized ~ 0.5–2 cm^3^ was placed into a tube containing phosphate-buffered saline (PBS) immediately after tumor resection. The specimen was pelleted in a benchtop centrifuge (Eppendorf, Germany) for 5 min at 100 *g* at 24°C. The tumor tissue was minced using a scalpel and treated with a mixture of enzymes: 0.5 mg of DNase (Sigma-Aldrich, USA), collagenase type I and type IV (1 mg each) (PanEco, Russia), and 2 mg of hyaluronidase (Microgen, Russia) in the handling medium (a 1 : 1 mixture of DMEM/F12 and RPMI 1640 media (PanEco, Russia), 2 mM *L*-alanyl-*L*-glutamine, 10% fetal bovine serum (FBS) HyClone (Cytiva, USA), supplemented with a 1× antibiotic–antimycotic solution (Gibco, USA)). The tube containing tissue fragments was placed on a rotating platform (Biosan, Latvia) and incubated at 7 rpm in a CO_2_ incubator at 37°C and 8% CO_2_ for 40 min. After incubation, the cell suspension was pipetted to break up aggregates and filtered through cell strainers with pore sizes of 100, 70, and 40 µm. The cells were pelleted in a benchtop centrifuge for 15 min (300 *g*, 24°C). The cell pellet was treated with 1 mL of ACK buffer (150 mM ammonium chloride, 10 mM potassium bicarbonate, 0.1 mM EDTA) for 1 min to lyse erythrocytes. The reaction was stopped with 2 mL of the handling medium. The cells were pelleted in a benchtop centrifuge for 7 min (300 *g*, 24°C ). The cell pellet was resuspended in 2 mL of Advanced DMEM (Thermo Fisher Scientific, USA) supplemented with 10% FBS HyClone (Cytiva), 2 mM *L*-alanyl-*L*-glutamine (Yeasen, China), and a 1× antibiotic–antimycotic solution (Gibco, USA). The isolated cells were cultured until the formation of colonies, which were cloned using cloning cylinders. The resulting primary tumor cultures were tested for sterility and mycoplasma contamination.

**Antibiotics and antimycotics**

The following stock solutions were used to optimize the antibiotic and antimycotic compositions: 50 mg/mL tylosin tartrate (Sigma-Aldrich) in water, 10 mg/mL tetracycline hydrochloride (Sigma-Aldrich) in 70% ethanol, 20 mg/mL ciprofloxacin hydrochloride (Sigma-Aldrich) in water, 50 mg/mL erythromycin (Sigma-Aldrich) in 95% ethanol, 34 mg/mL chloramphenicol (Thermo Fisher Scientific, USA) in 95% ethanol, 5 mg/mL tigecycline (Thermo Fisher Scientific, USA) in DMSO, 20 mg/mL moxifloxacin hydrochloride (Thermo Fisher Scientific, USA) in DMSO, 20 mg/mL levofloxacin (Sigma-Aldrich) in DMSO, 50 mg/mL voriconazole (Sigma-Aldrich) in DMSO, 50 mg/mL colistin sulfate (Thermo Fisher Scientific, USA) in water, 20 mg/mL polymyxin B sulfate (Sigma-Aldrich) in water, 5 mg/mL eremomycin sulfate (Gause Institute of New Antibiotics, Moscow, Russia) in water, 50 mg/mL vancomycin hydrochloride (Thermo Fisher Scientific, USA) in water, 50 mg/mL micafungin sodium (Sigma-Aldrich) in water, and 50 mg/mL meropenem trihydrate (Thermo Fisher Scientific, USA) in DMSO. All the solutions were subjected to sterile filtration through a filter (pore size, 0.22 µm) prior to application. The solvents (DMSO and ethanol (Thermo Fisher Scientific, USA)) were used without additional purification. The solutions of antimicrobials were prepared using Milli-Q water (Merck Millipore, Germany).

**Mycoplasma eradication and prevention of primary cell culture contamination**

Cell lines and primary cell cultures were cultivated in Advanced DMEM (Thermo Fisher Scientific, USA) supplemented with 10% FBS HyClone (Cytiva), 2 mM *L*-alanyl-*L*-glutamine (Yeasen, China), and a 1× antibiotic–antimycotic solution (Gibco, USA).

An antibiotic composition (5 µg/mL tylosin, 5 µg/mL tetracycline, 20 µg/mL ciprofloxacin, 20 µg/mL chloramphenicol, and 50 µg/mL erythromycin) was added to the cell cultures to achieve mycoplasma eradication. Complete eradication of mycoplasma from the cell lines was observed after 4 weeks of cultivation involving 7–9 passages.

The following antibiotics were also added to prevent contamination of primary tumor cell cultures when isolating tumor cells from surgical specimens and upon cultivation: 1 µg/mL levofloxacin, 20 µg/mL polymyxin B, 20 µg/mL vancomycin, 20 µg/mL meropenem, 10 µg/mL micafungin, and 10 µg/mL voriconazole. This antibiotic–antimycotic composition was added until the stages of cryopreservation and generation of master cell banks.

**Cytotoxicity testing**

The cytotoxicity of individual antibiotics and their combination was evaluated using HEK-293T cells cultivated in DMEM (Thermo Fisher Scientific, USA) supplemented with 10% FBS HyClone, 2 mM *L*-alanyl-*L*-glutamine, and a 1× antibiotic–antimycotic solution. Cell viability was measured by colorimetry in the methyl tetrazolium (MTT) assay used to determine cellular metabolic activity. Twofold dilutions of test compounds in the growth medium were prepared to conduct the assay. The antibiotics were tested starting at a concentration of 200 µg/mL for individual compounds or from a 16- to 0.03-fold concentration of an antibiotic mixture. The assay was carried out after incubating cells in the growth medium containing the drug solutions in a humidified atmosphere at 37°C with 8% CO_2_ for 72 h, at an initial cell confluence of 85%. Next, an MTT solution was added to the test samples (10 µL per well) to a final concentration of 0.45 mg/mL. Incubation at 37°C was conducted for 1 h. A solubilizing solution (100 µL) was then added to each well to dissolve formazan crystals. The solubilizing solution was prepared as an aqueous mixture of dimethylformamide (40 vol/vol %) and glacial acetic acid (2 vol/vol %). The resulting solution was used to dissolve sodium dodecyl sulfate (16 wt/vol %), and the pH was adjusted to 4.7. The absorbance was measured using a Varioskan multimode microplate reader (Thermo Fisher Scientific, USA) at 570 nm. The cytotoxic concentration (IC_50_) was determined from the resulting values using Prism 9.1.1 software (GraphPad, USA).

**Preparing cell culture supernatant samples for the mycoplasma detection test**

The cells were cultivated in DMEM medium supplemented with 10% FBS HyClone, 2 mM *L*-alanyl-*L*-glutamine, and a 1× antibiotic–antimycotic solution for 3 days. By the test day, the cells had reached 80–90% monolayer confluence. The culture medium (1 mL) was aliquoted. The cells and cell debris were precipitated by centrifugation at 500 *g* for 5 min. The supernatant was transferred into new tubes and centrifuged at 15,000 *g* for 10 min. The supernatant was then carefully removed without disturbing the pellet. PBS (50 µL) was added to the pellet, and the mixture was resuspended thoroughly. The tubes were heated at +95°C for 10 min, and the resulting solutions were used for PCR amplification.

**Mycoplasma detection test**

A MycoReport kit (Evrogen, Russia) was used to detect mycoplasma contamination in the cell lines. Each PCR run included two control samples: a positive control (C+) and a negative control without a template (C-). All samples, including the controls, were tested in two reactions: MR (the target reaction) and MC (internal PCR control). The amount of the sample added to each reaction was 2 µL.

PCR amplification was performed according to *Table 1*. The samples were then analyzed by agarose gel electrophoresis (1%).

**Table 1:** PCR amplification protocol

Temperature, °C	Stage	Incubation time	Number of cycles
+37	UDG treatment	10 min	1
+95	Pre-denaturation	10 min	1
+95	Denaturation	15 s	35
+62	Annealing	15 s	
+72	Elongation	15 s	

## RESULTS AND DISCUSSION

**Mycoplasma eradication**

Mycoplasma is the most common microorganism responsible for cell culture contamination. Because of the small size of mycoplasma cells and the intracellular nature of the infection, mycoplasma contamination is less readily detected, and it is primarily diagnosed using PCR-based techniques. To achieve mycoplasma eradication, cells need to be cultivated in the presence of antimicrobial agents for a long time, making this approach reasonable only for cell cultures of exceptional value.

Commercial agents for mycoplasma elimination are not always effective or their efficacy and toxicity profiles are suboptimal [17]. To develop an optimal antibiotic composition for mycoplasma eradication, we focused on antibiotics that are highly potent against various mycoplasma strains (fluoroquinolones, macrolides, and tetracyclines) and those exhibiting low resistance rates in different mycoplasma strains (14% tetracycline, 15% ciprofloxacin, 21% tylosin, and 30% chloramphenicol) [13]. To optimize the antibiotic composition, we assessed the cytotoxicity of antimicrobials of different classes: macrolides (tylosin and erythromycin), fluoroquinolone (ciprofloxacin), tetracycline, and chloramphenicol (*Fig. 1*).

**Fig. 1:**
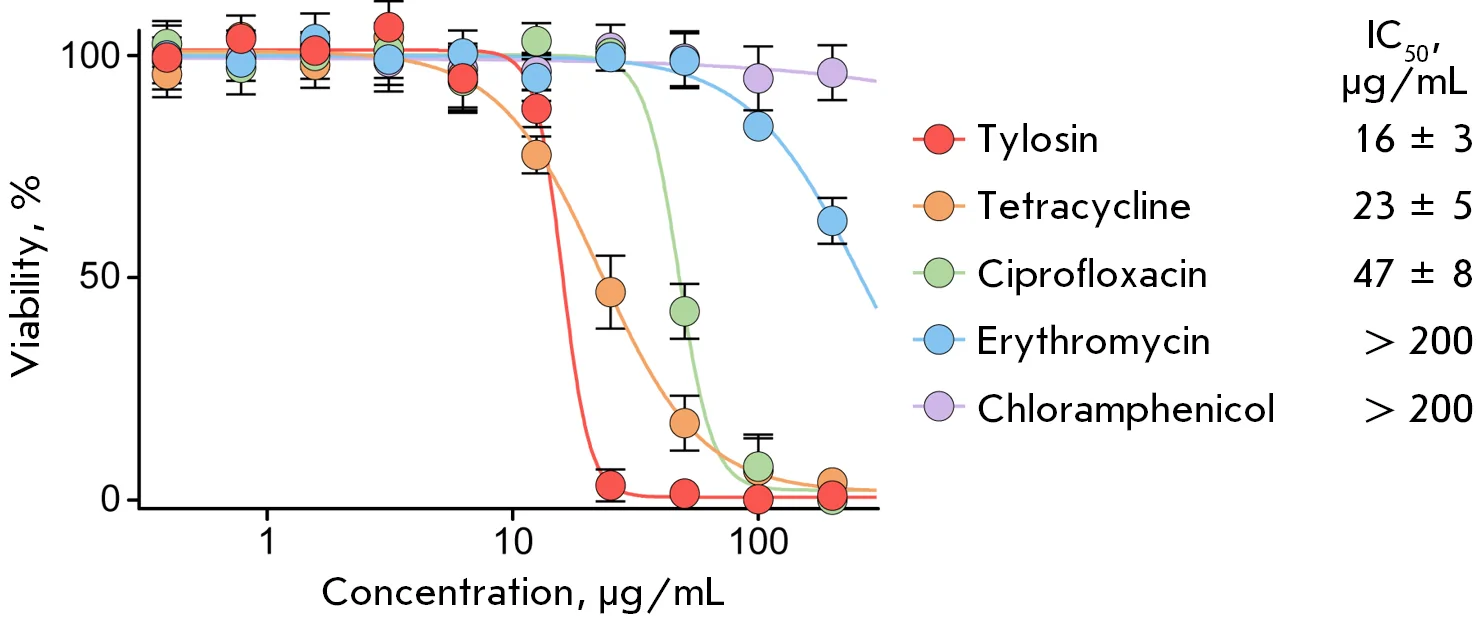
Cytotoxicity of highly effective anti-mycoplasma agents in human HEK-293T cells. Data were obtained from three independent biological replicates; error bars represent standard deviations

The final concentrations of antimicrobial agents were selected according to the cytotoxicity curves of the individual components of the combination of antimicrobials so as to minimize their effect on the proliferative activity of cell cultures. Among the selected anti-mycoplasma agents, significant toxicity was observed for tylosin (IC_50_ = 16 ± 3 µg/mL), tetracycline (IC_50_ = 23 ± 5 µg/mL), and ciprofloxacin (IC_50_ = 47 ± 8 µg/mL). Meanwhile, these concentrations are 1.5–2 orders of magnitude higher than the mean minimal inhibitory concentrations for mycoplasma strains, thus enabling their efficient use in laboratory practice. A combination of all five antibiotics at a tylosin : tetracycline : ciprofloxacin : chloramphenicol : erythromycin ratio = 1 : 1 : 4 : 4 : 10 was used to maximize the synergistic effect. The ratio between the concentrations was selected in accordance with the cytotoxicity of the antibiotics: the concentration of the fluoroquinolone, a bactericidal agent, was relatively increased, while the concentration of chloramphenicol was reduced because of its limited solubility in water. The optimization of antibiotic concentrations in this combination using HEK-293T cells demonstrated that > 95% cell viability was achieved by using the following final concentrations: 5 µg/mL tylosin; 5 µg/mL tetracycline; 20 µg/mL ciprofloxacin; 20 µg/mL chloramphenicol; and 50 µg/mL erythromycin (*Fig. 2*).

**Fig. 2:**
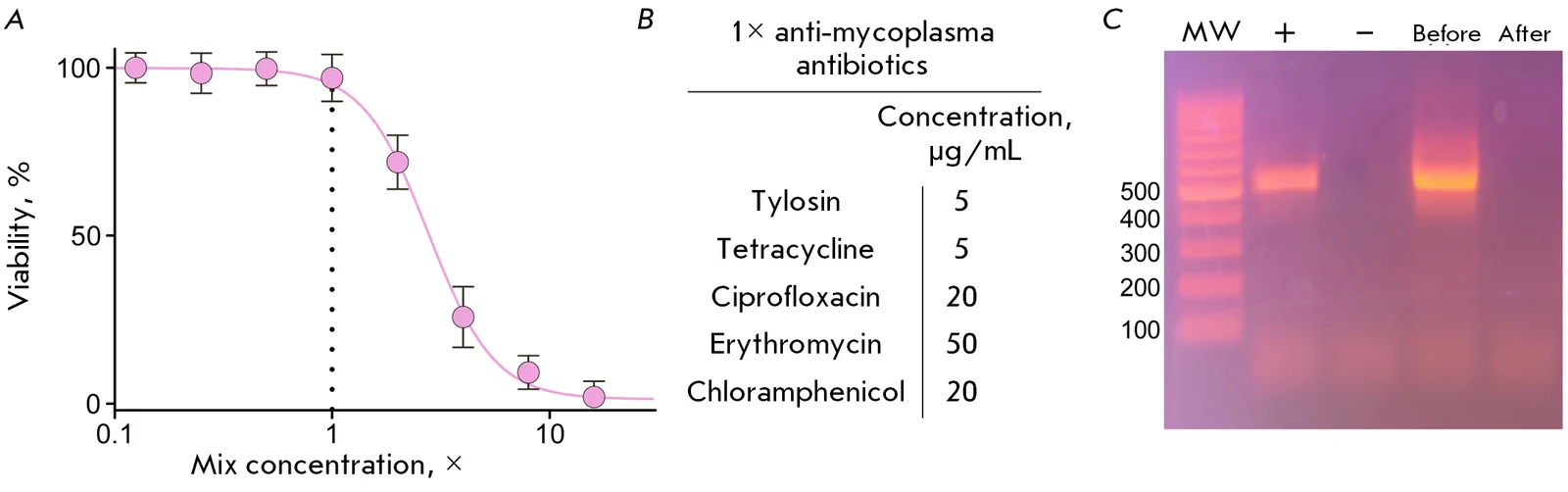
Optimization of the concentrations for the combination of anti-mycoplasma agents. (*A*) Cytotoxicity of the anti-mycoplasma agent mixture in human HEK-293T cells. Data were obtained from three independent biological replicates; error bars represent the standard deviations. (*B*) Composition and concentrations of antibiotics in the 1× mixture. (*C*) PCR test for the detection of mycoplasma contamination. MW is a molecular weight marker; “+” and “–” are positive and negative control samples, respectively; the HEK-293T cell culture contaminated with mycoplasma before cultivation with antibiotics (Before) and after cultivation with antibiotics (After) is shown

The effectiveness of the selected antibiotic combination was tested on a panel of different mycoplasma-contaminated human cell lines, including HEK-293T, A172, A431, U87, U251, HT-1080, and NCI-H1299. It was demonstrated that four weeks of cultivation involving 7–9 passages in a growth medium containing the 1× combination of anti-mycoplasma agents ensured complete elimination of mycoplasma (7/7 cell lines); mycoplasma contamination was detected in none of the cell lines even after an additional 8–10 passages in an antibiotic-free growth medium (*Fig. 2C*).

**Prevention of primary cell culture contamination with bacteria and fungi**

Isolation of mammalian microbiome-associated cells is particularly challenging because of the risk of bacterial and fungal contamination. The conventional antibiotic–antimycotic mixtures (Gibco) are often ineffective for the isolation of primary tumor cell cultures from colorectal cancer patients. Thus, more than 60% of the isolates (6/9) became contaminated with microorganisms when the Gibco 1× antibiotic–antimycotic mixture was used. Both bacterial (4/6) and fungal (2/6) contamination were detected.

An antibiotic and antimycotic composition including the most effective therapeutic agents displaying broad- and narrow-spectrum activity was designed to prevent microbial contamination. To optimize this composition, we assessed the cytotoxicity of antibiotics of different classes (fluoroquinolones, lipopeptides, glycopeptides, carbapenems, and tetracyclines) displaying different antibacterial spectra of activity (polymyxins for the eradication of Gram-negative microorganisms and glycopeptides for Gram-positive ones). Furthermore, the combination also comprised the highly potent antimycotics voriconazole and micafungin (*Fig. 3*).

**Fig. 3:**
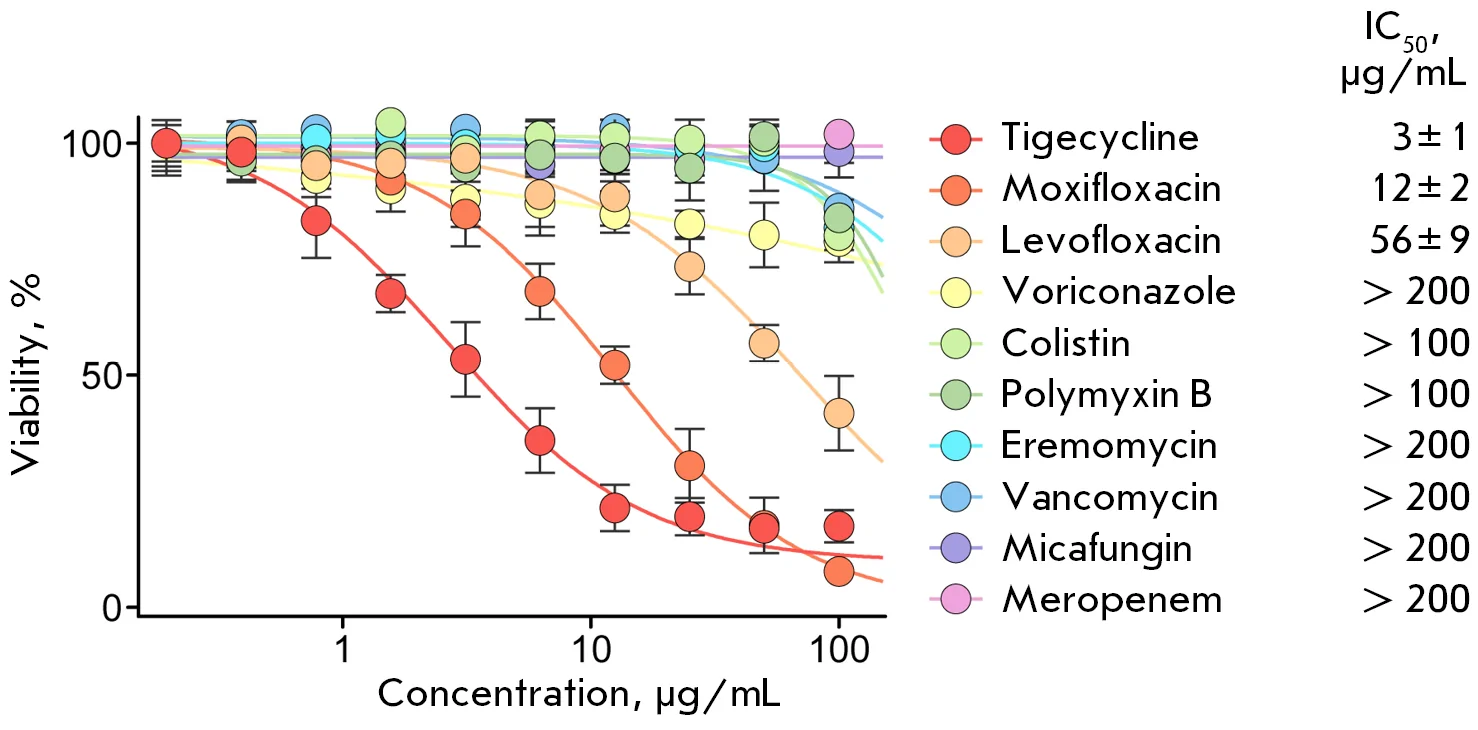
Cytotoxicity of highly effective agents for the prevention of bacterial and fungal contamination of primary cell cultures toward human HEK-293T cells. Data were obtained from three independent biological replicates; error bars represent standard deviations

Among the selected therapeutic agents, significant toxicity was observed for tigecycline (IC_50_ = 3 ± 1 µg/mL), moxifloxacin (IC_50_ = 12 ± 2 µg/mL), and levofloxacin (IC_50_ = 56 ± 9 µg/mL). Therefore, tigecycline was excluded from further consideration because of its high cytotoxicity, while polymyxin B, colistin, eremomycin, and vancomycin displayed low cytotoxicity and high efficacy profiles. Fluoroquinolones were the most toxic component of the combination. However, they were included in the combination because these agents are highly effective broad-spectrum antibiotics characterized by relatively low resistance in bacteria. The upper bounds of the minimal inhibitory concentrations for antibiotics associated with the intermediate level of resistance in bacteria (~20 µg/mL) and fungi (~10 µg/mL) were used for preventing primary culture contamination. The combination consisting of 20 µg/mL polymyxin B, 20 µg/mL vancomycin, 20 µg/mL meropenem, 10 µg/mL micafungin, and 10 µg/mL voriconazole was selected as the basal combination of antibacterial and antifungal agents. In order to optimize the concentrations of therapeutic agents and select a particular fluoroquinolone in the combination of antimicrobials, we evaluated the effect of fluoroquinolone and mix concentrations on the cytotoxicity of the combination of agents (*Fig. 4*)*.*

**Fig. 4:**
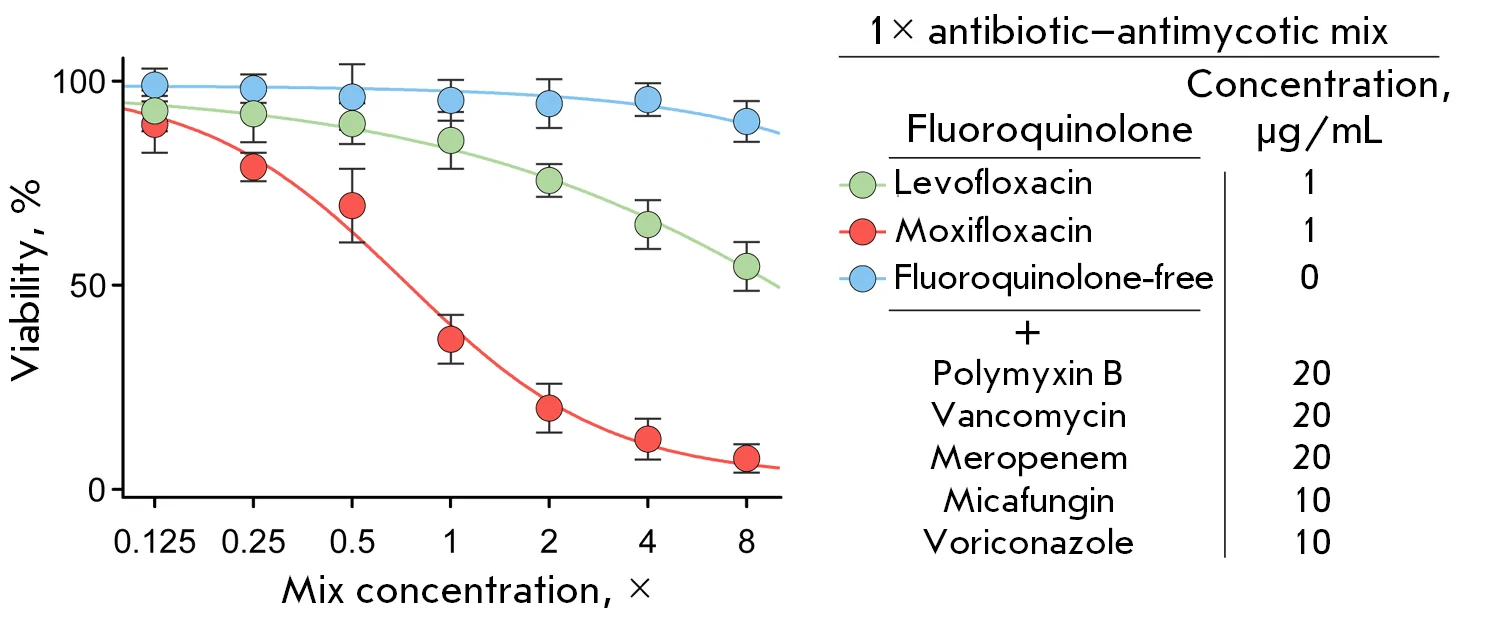
Optimization of the concentration for the compositions to prevent bacterial and fungal contamination of primary cell cultures. Cytotoxicity of various concentrations and combinations in human HEK-293T cells. Data were obtained from three independent biological replicates; error bars represent standard deviations

The higher cytotoxicity of moxifloxacin largely compensates for its potentially higher antimicrobial activity compared to that of levofloxacin, thus limiting its use in this combination. Meanwhile, the combination containing 1 µg/mL levofloxacin yielded more than 85% cell viability; cell viability for the composition containing no fluoroquinolone was more than 95% (*Fig. 4*). This composition, with 1 µg/mL levofloxacin added, prevented contamination of primary tumor cell cultures isolated from colorectal cancer patients (0/34) without dramatically reducing the cell growth rate.

## CONCLUSION

Laboratory-derived contamination of cell lines is largely the result of improper cell culture practices. If contamination is detected, it is imperative that all contaminated biospecimens be eliminated and all laboratory equipment related to them be subjected to thorough cleaning and sterilization. Meanwhile, in special situations, a specimen under analysis can be unique and irreplaceable. It is clear that indiscriminate antibiotic use is absolutely unacceptable. Nevertheless, the recovery of unique contaminated biospecimens can be considered reasonable if a biospecimen holds exceptional value. Moreover, the procedure used to isolate microbiome-associated biospecimens, especially those associated with the gut microbiome, comes with a high risk of contamination. Conventional contamination control agents lack effectiveness, which is often a result of the spread of antibiotic resistance.

The approaches proposed in this study are primarily intended for work with unique biospecimens that either cannot be replicated or the cost of the replication of which is prohibitively high (*Fig. 5*).

**Fig. 5:**
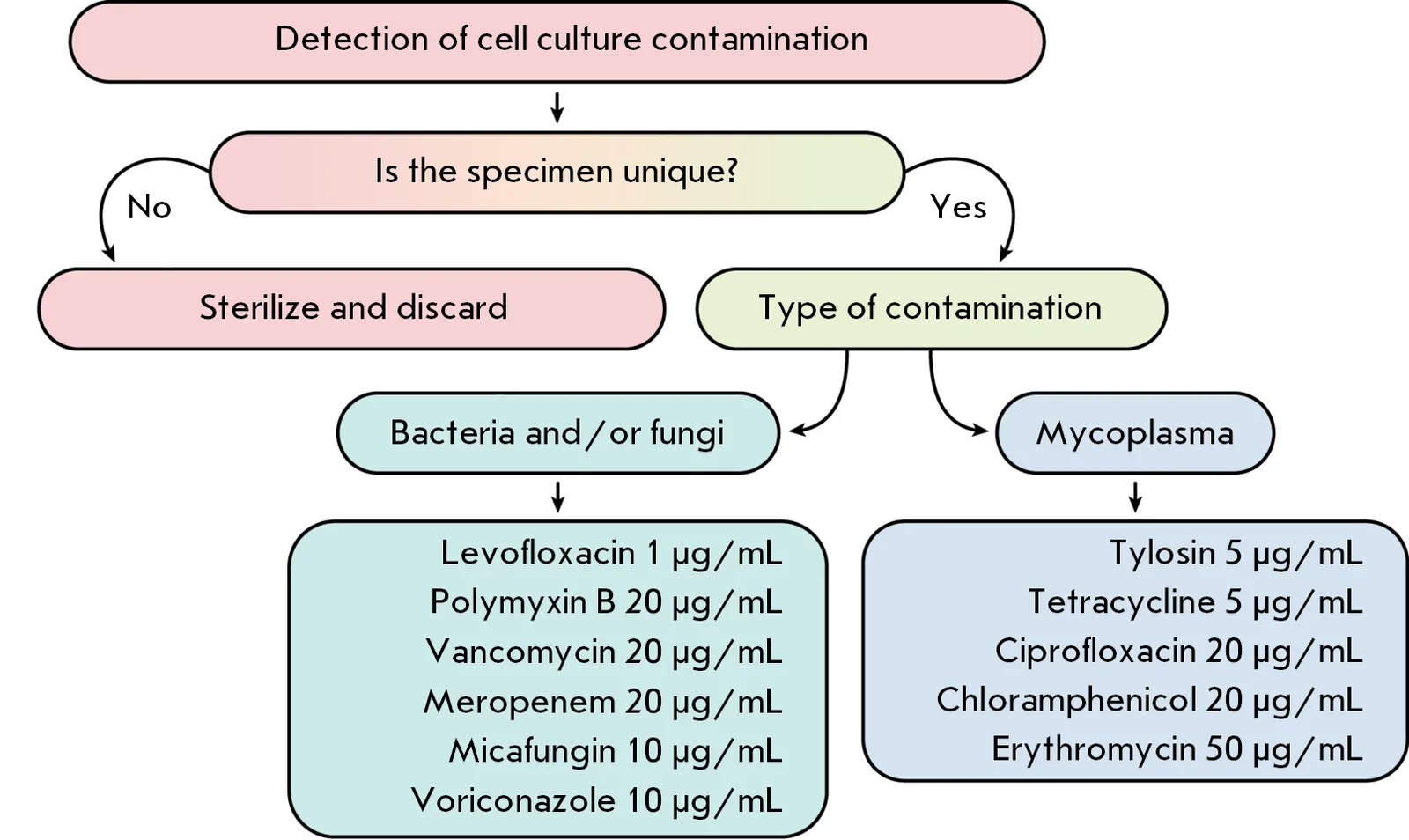
Decision-making strategy for working with contaminated cell cultures and the compositions of antimicrobial agents for decontamination, depending on the type of contamination

The key factor in the selection of antibiotic combinations is broadening their spectrum of activity. For mycoplasma infection, the choice of antibiotics is primarily predicated on a low prevalence of resistant strains and high antibacterial activity. In turn, the most effective broad-spectrum antibiotics (third- and fourth-generation fluoroquinolones and meropenem), supplemented with highly potent antibiotics against Gram-negative (polymyxin B) and Gram-positive (vancomycin) antimicrobial-resistant bacteria, as well as the most effective antifungals (micafungin and voriconazole), were selected to design the composition to prevent contamination of primary cell cultures isolated from surgical resections of colorectal cancer patients. These compositions enabled complete mycoplasma eradication and prevented contamination during primary cell culture isolation.

Importantly, fluoroquinolones are the most toxic components of the compositions; so, they can potentially be excluded from consideration (in the case of levofloxacin), or their concentrations can be reduced (two- to fourfold in the case of ciprofloxacin). Nonetheless, a more rational strategy would be to reduce the concentrations of the developed combinations. Meanwhile, this makes sense only if the selected cell line or cell culture displays extraordinary sensitivity, since irreversible toxicity was detected for none of the analyzed cell lines (0/7) or primary cultures (0/34) in this study. Another point is that antibiotics, and fluoroquinolones in particular, may induce phenotypic transformation in cell lines. As repeatedly mentioned above, the protocols reported in this study should be considered as a last resort. Both exposure of cell lines to antibiotics and their clinical application in general should be minimized. The developed approaches allow one to preserve unique biospecimens and decontaminate complex biospecimens of exceptional scientific value. This expands the potential of cell biology pipelines and lays the necessary groundwork for functional studies, including those employing unique biospecimens associated with antibiotic-resistant microbiomes.
